# Soft and Hard Tissue Stability Following Guided Bone Regeneration in an Active Orthodontics Patient

**DOI:** 10.7759/cureus.77496

**Published:** 2025-01-15

**Authors:** Rusmizan Yahaya, Nurul Ain Mohamed Yusof, Muhammad Annurdin Sabarudin

**Affiliations:** 1 Periodontology, Kota Setar Dental Specialist Clinic, Ministry of Health, Alor Setar, MYS; 2 Periodontology, Kota Setar Dental Specialist Clinic, Alor Setar, MYS; 3 Periodontology, Universiti Sains Islam Malaysia, Kuala Lumpur, MYS

**Keywords:** acellular dermal matrix, barrier membrane, guided bone regeneration, orthodontic treatment, periodontal surgery

## Abstract

Guided bone regeneration (GBR) is a bone augmentation technique widely used in clinical practice to promote new bone growth by creating a conducive environment through bone grafts and barrier membranes. This article reports a case of a 14-year-old patient with significant bone loss, wound breakdown, and gingival recession in the anterior maxillary tooth, which was successfully treated through GBR combined with orthodontic treatment. This complex case of an area with bone defect required a multidisciplinary treatment in order to provide the best treatment for young patients.

## Introduction

Guided bone regeneration (GBR) has become a cornerstone procedure in periodontics and implant dentistry, especially in cases involving significant bone loss that impacts both function and aesthetics [1]. GBR aims to promote new bone growth by creating a conducive environment through the use of bone grafts and barrier membranes, which help stabilize the graft material and prevent soft tissue invasion [2]. In complex cases involving orthodontic treatment, GBR can present unique challenges due to the constant movement and remodeling of periodontal tissues, making surgical planning and timing crucial to achieving favorable outcomes [3,4].

In orthodontic cases with existing bone and soft tissue deficiencies, managing the delicate balance between bone regeneration and orthodontic movement is essential. The choice of biomaterials, surgical techniques, and timing of force application must be carefully considered to optimize the long-term stability of the periodontium and ensure a successful treatment outcome.

The case emphasizes the importance of a multidisciplinary approach, integrating periodontal and orthodontic care to address complex anatomical and aesthetic needs. By employing GBR alongside frenotomy and vestibuloplasty, the surgical team aimed to create a stable and tension-free environment, allowing for adequate bone regeneration and soft tissue preservation. This case report highlights the procedural strategies, materials used, and timing considerations in the context of achieving optimal soft and hard tissue stability, ultimately contributing to both periodontal health and orthodontic success in a young patient.

## Case presentation

**Figure 1:**
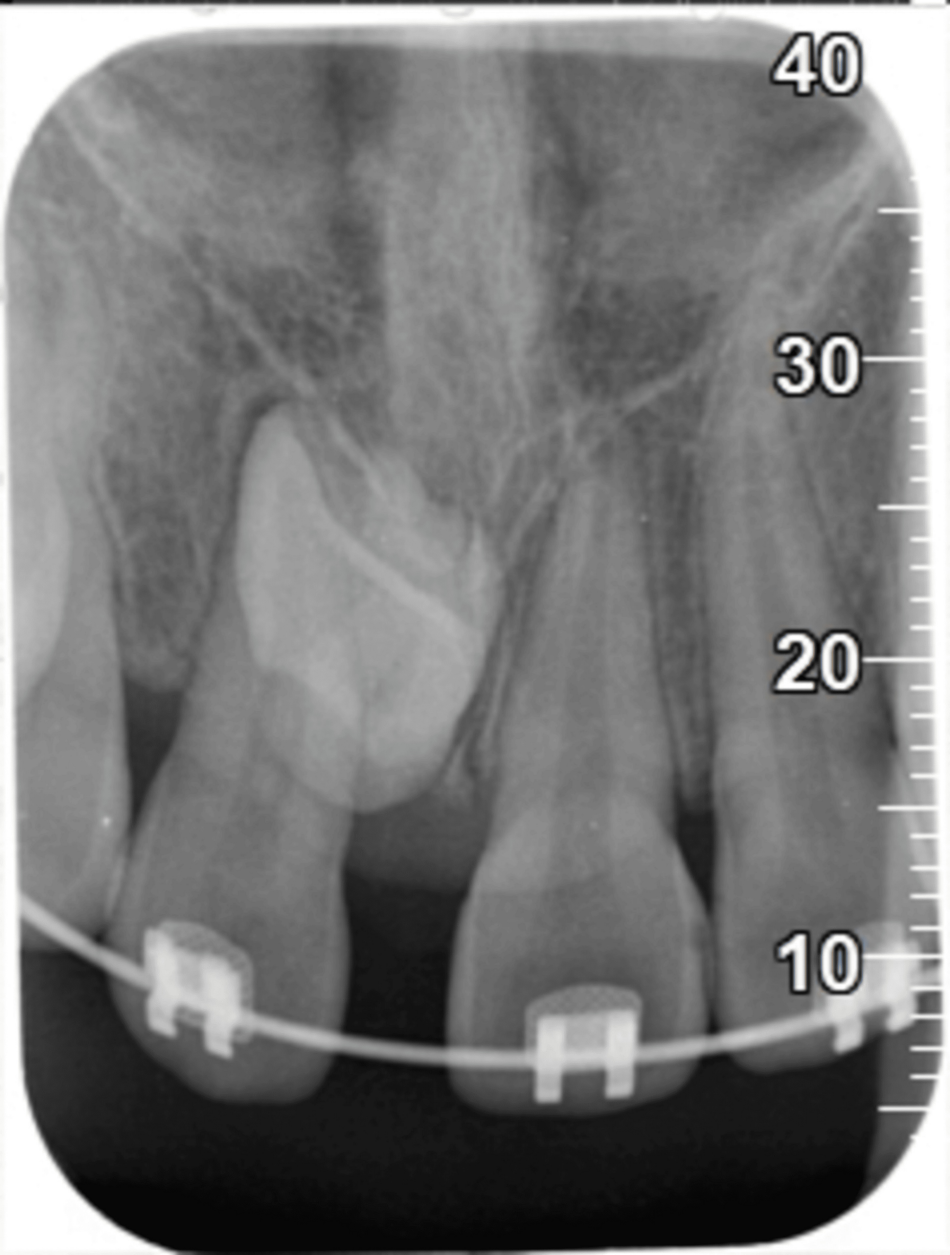
Periapical radiograph revealed impacted tooth 11.

A 14-year-old female patient was referred to the Periodontics Specialist Unit, Kota Setar Specialist Dental Clinic, for the management of significant bone loss with wound breakdown in 11 areas and gingival recession of tooth 12. This patient was initially managed by orthodontic and pediatric specialists with a history of closed technique of surgical exposure and gold chain placement on teeth 11 and 12. After two years postoperatively, tooth 11 was noted to be impacted with dilaceration root, which led to an irrational treatment prognosis (Figure 1). Surgical removal of impacted tooth 11 without bone grafting was performed under general anesthesia (GA) by the pediatric team.

**Figure 2:**
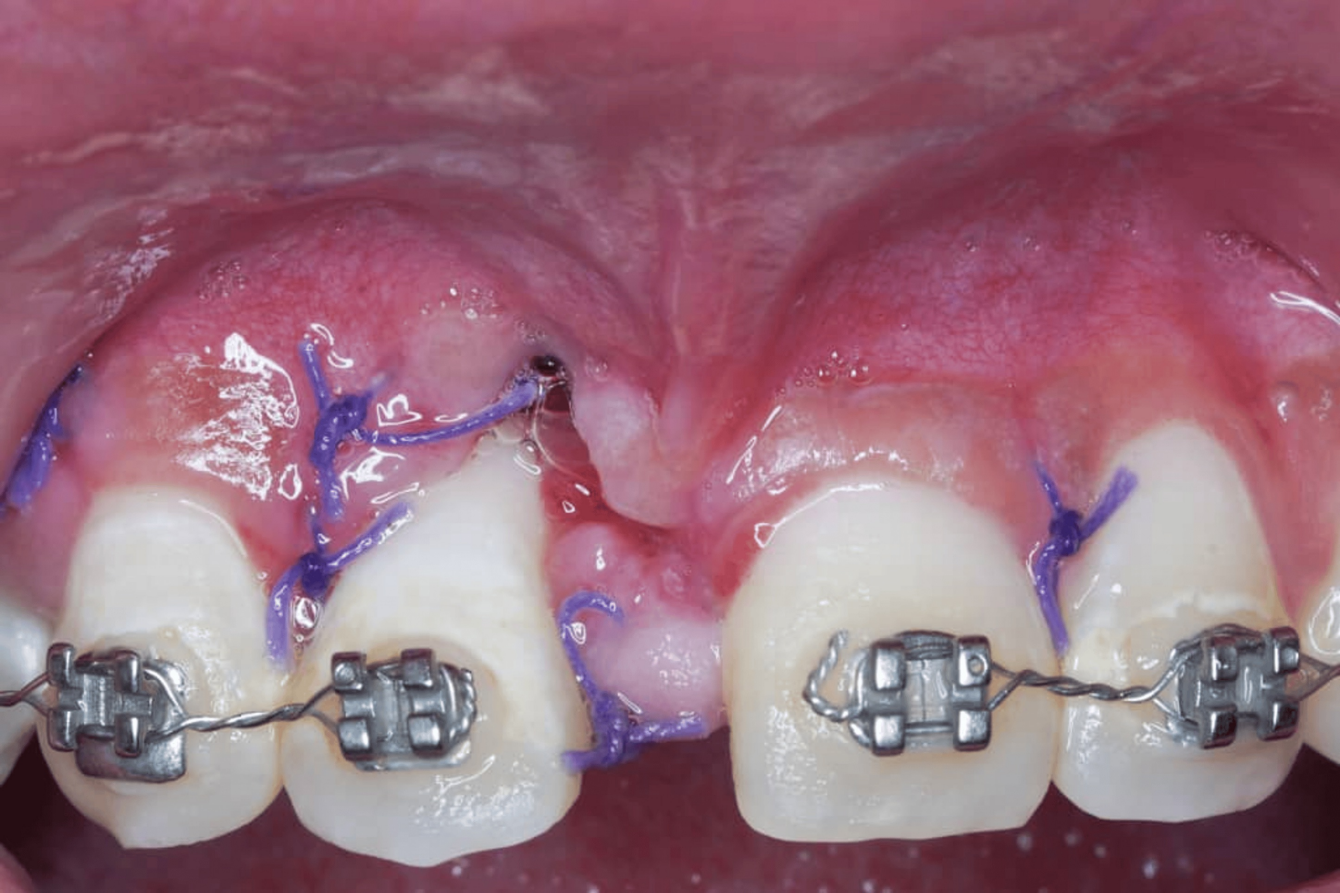
Baseline photograph shows 2 weeks post-surgical extraction of tooth 11 with wound breakdown and gingival recession of mesial 12.

**Figure 3:**
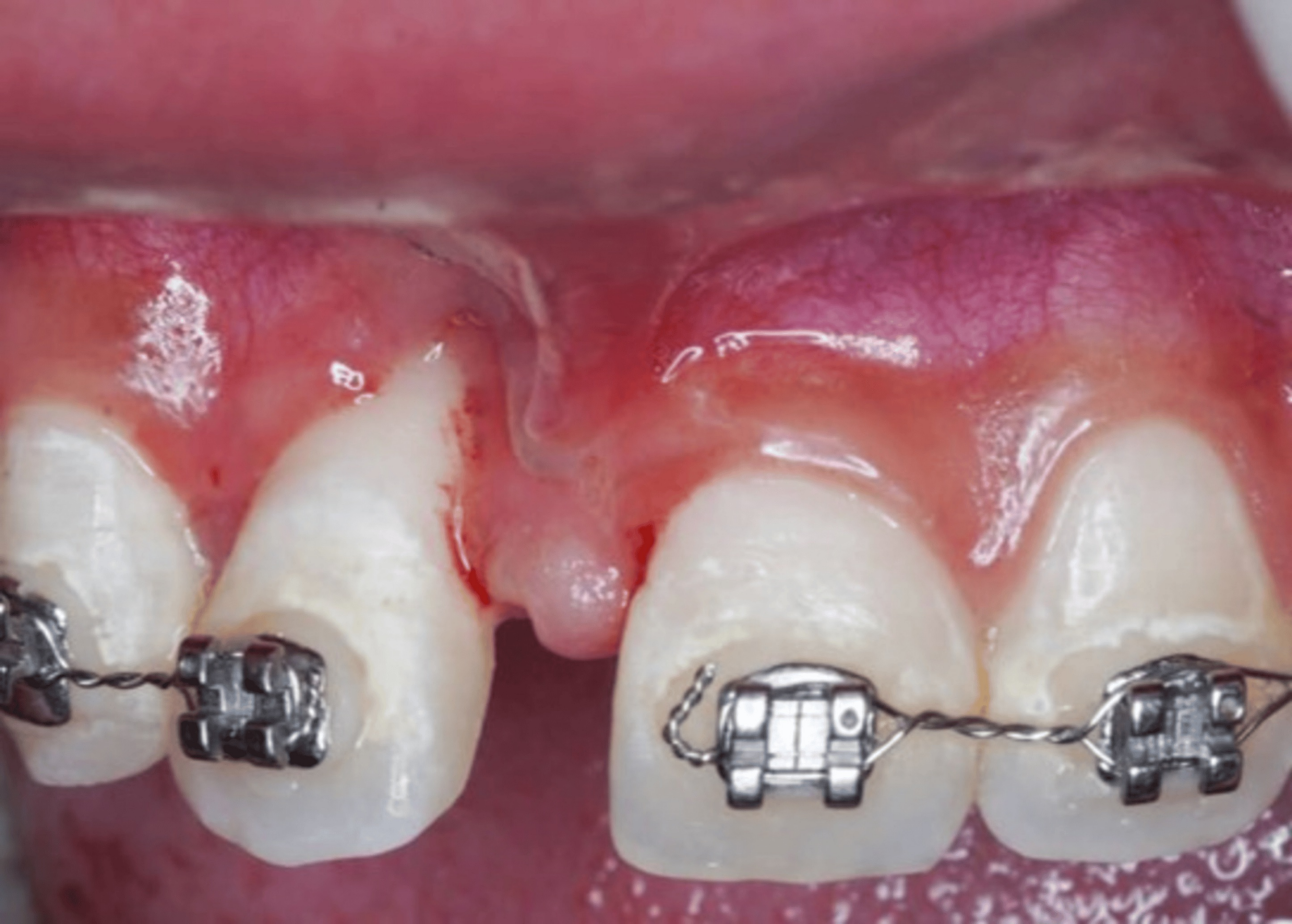
Pre-op photograph after 6 weeks of non-surgical periodontal therapy.

**Figure 4:**
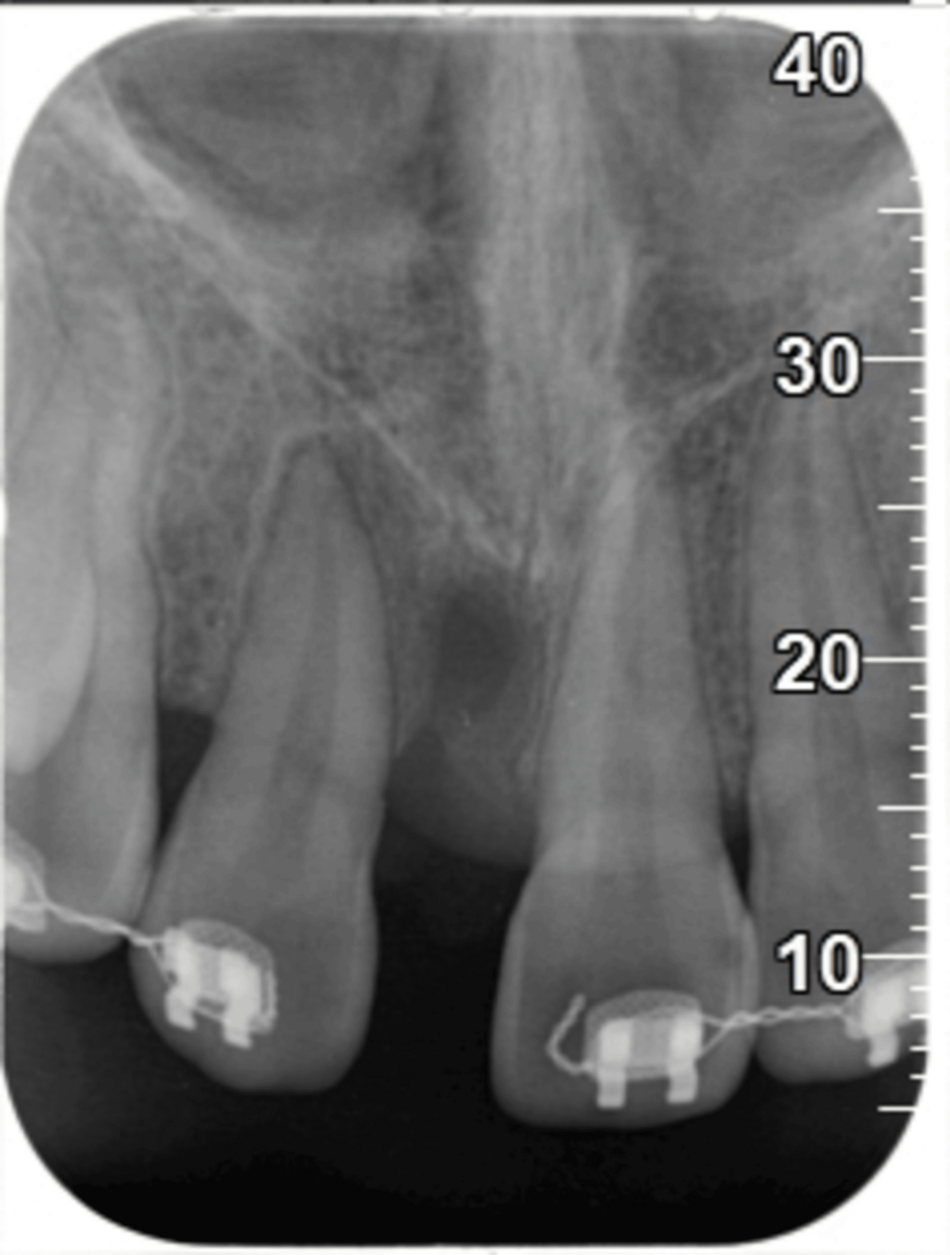
Periapical radiograph revealed a crater bone defect at the surgical extraction of 11.

On the day of the GBR surgery, the surgical procedure was explained to the patient and her mother, and written consent was taken. The patient was rinsed with chlorhexidine gluconate 0.12%, and local anesthesia was administered using lidocaine 2% with epinephrine 1:100,000. Subsequently, a full-thickness mucoperiosteal flap was elevated with intrasulcular incisions from teeth 12 until 22 and simplified papilla preservation flap between interdental 13 and 12; 21 and 22 to avoid recession and interdental loss on these teeth.

**Figure 5:**
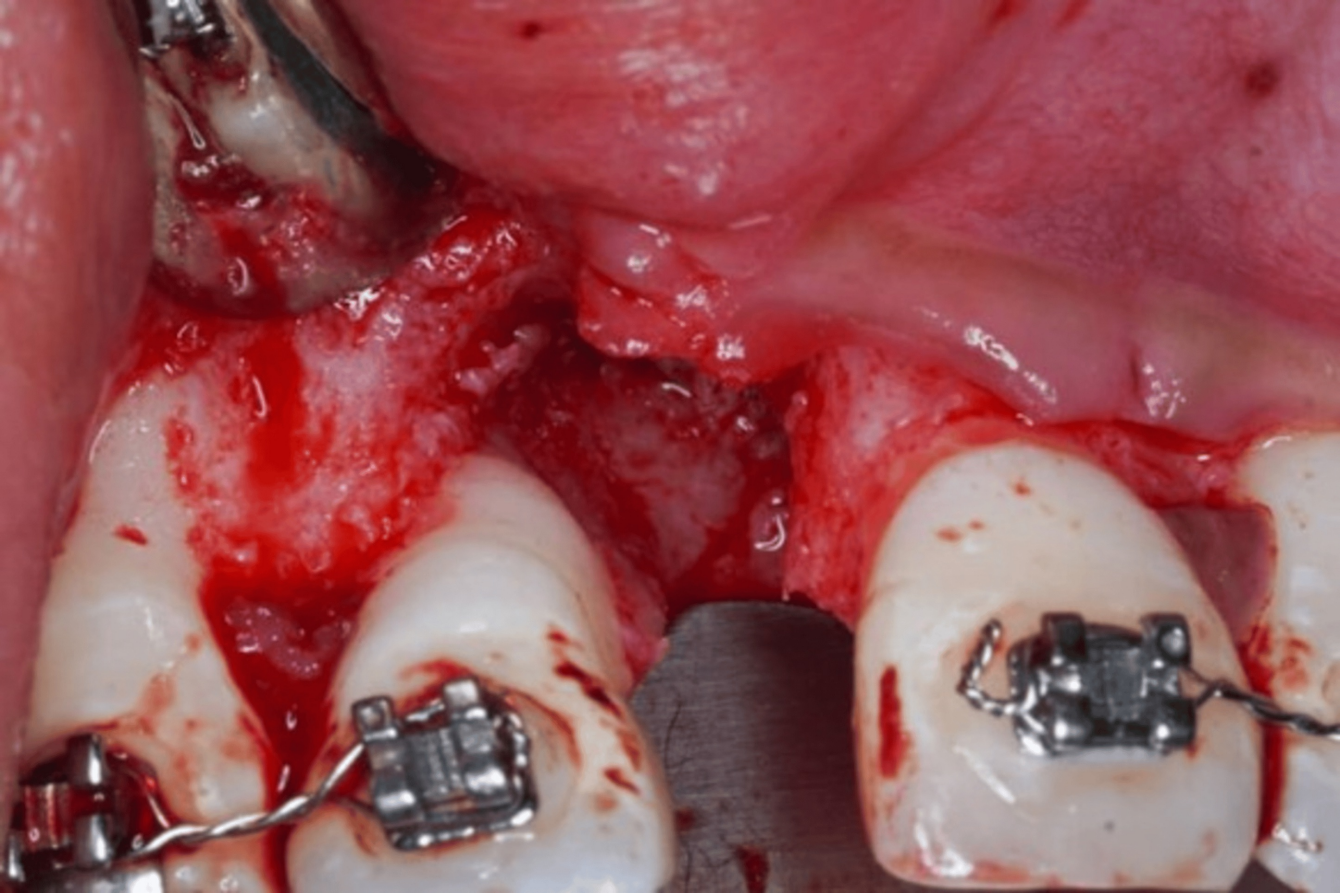
Following full-thickness flap elevation, a significant interdental bone loss on mesial 12 presented.

**Figure 6:**
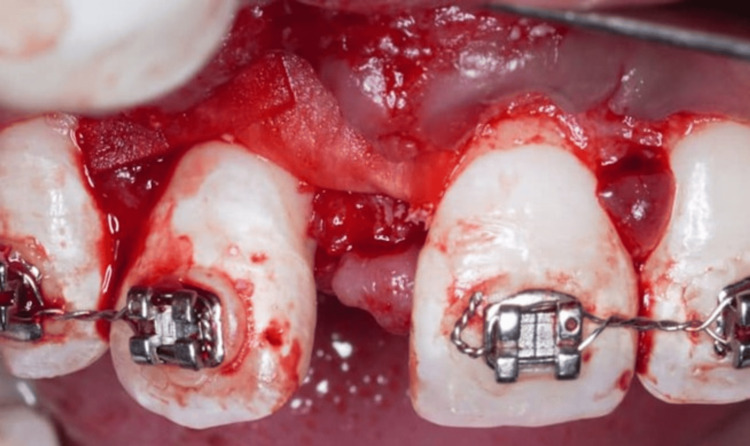
Placement of bovine-derived xenograft of 0.25 g and acellular dermal matrix membrane.

Following the degranulation of soft tissue, a significant interdental bone loss was presented on the mesial of tooth 12 (Figure 5). A bovine-derived xenograft of 0.25 g and an acellular dermal matrix (ADM) membrane were placed on the defect (Figure 6). Furthermore, frenotomy and deepening of sulcus/vestibuloplasty were performed subsequently. Consequently, the labial flap was sutured by means of a 4/0 polytetrafluoroethylene (PTFE) suture using a modified horizontal mattress (Laurel’s technique) and a simple interrupted suture to achieve primary closure of the surgical wound. Additionally, postoperative instructions, including oral hygiene instructions, were provided.

**Figure 7:**
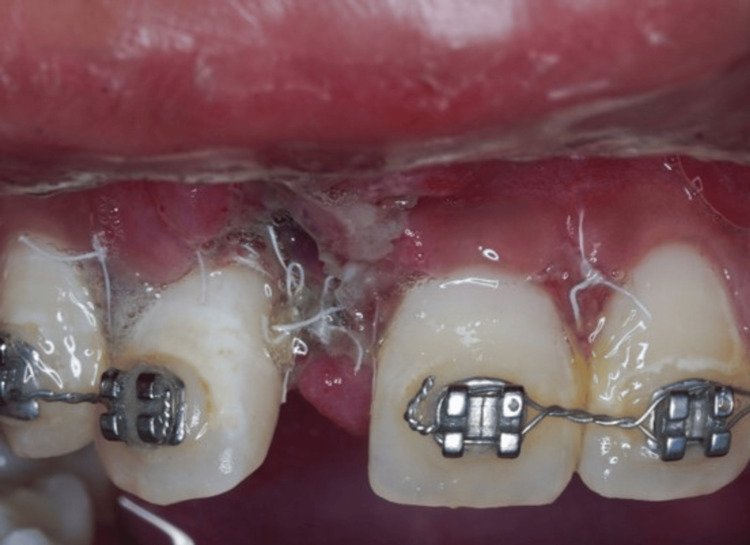
Postoperative photograph shows uneventfully healed surgical site (1-week post-op).

**Figure 8:**
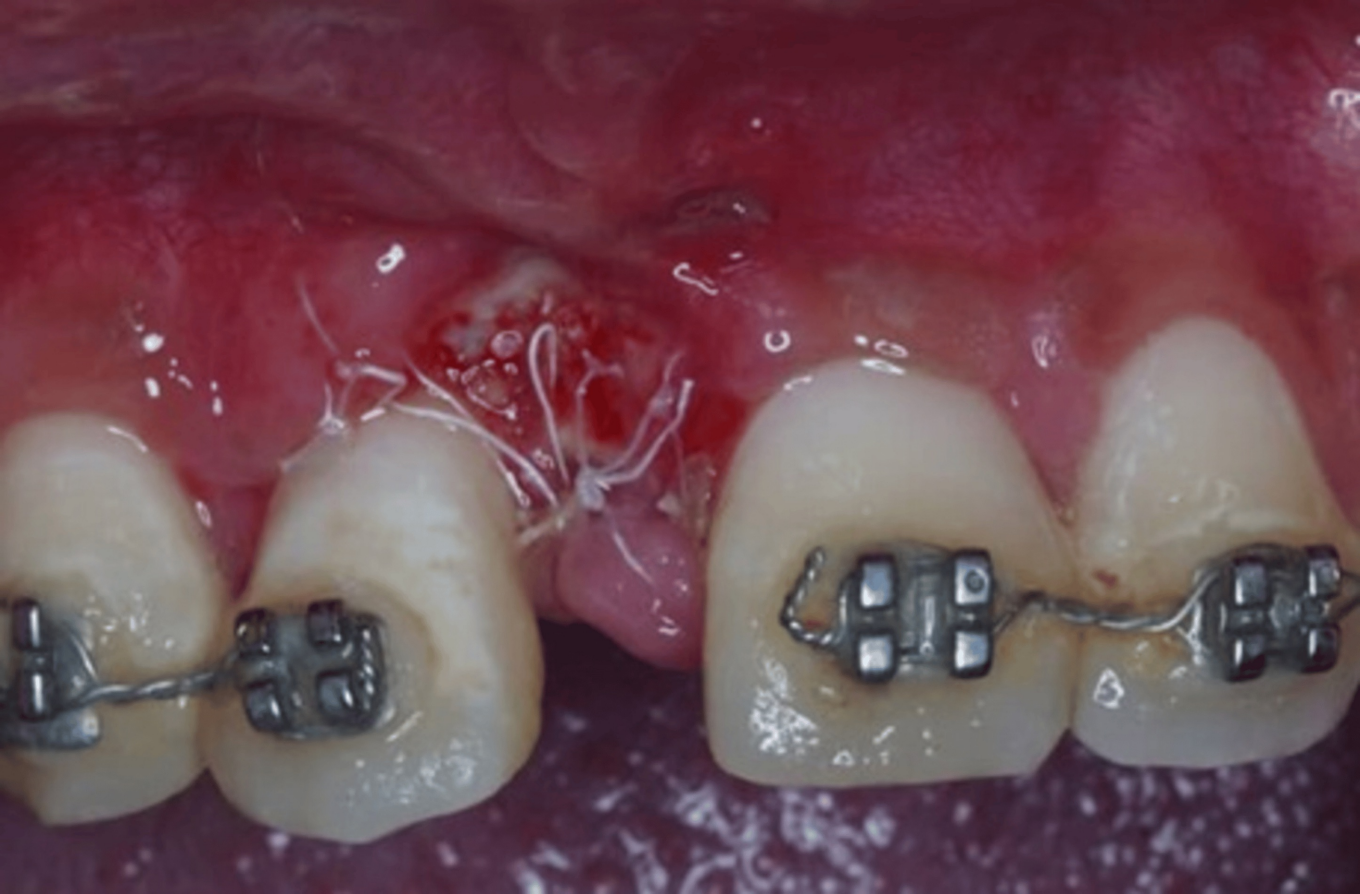
Postoperative photograph shows clinical presentation (2-week post-op).

**Figure 9:**
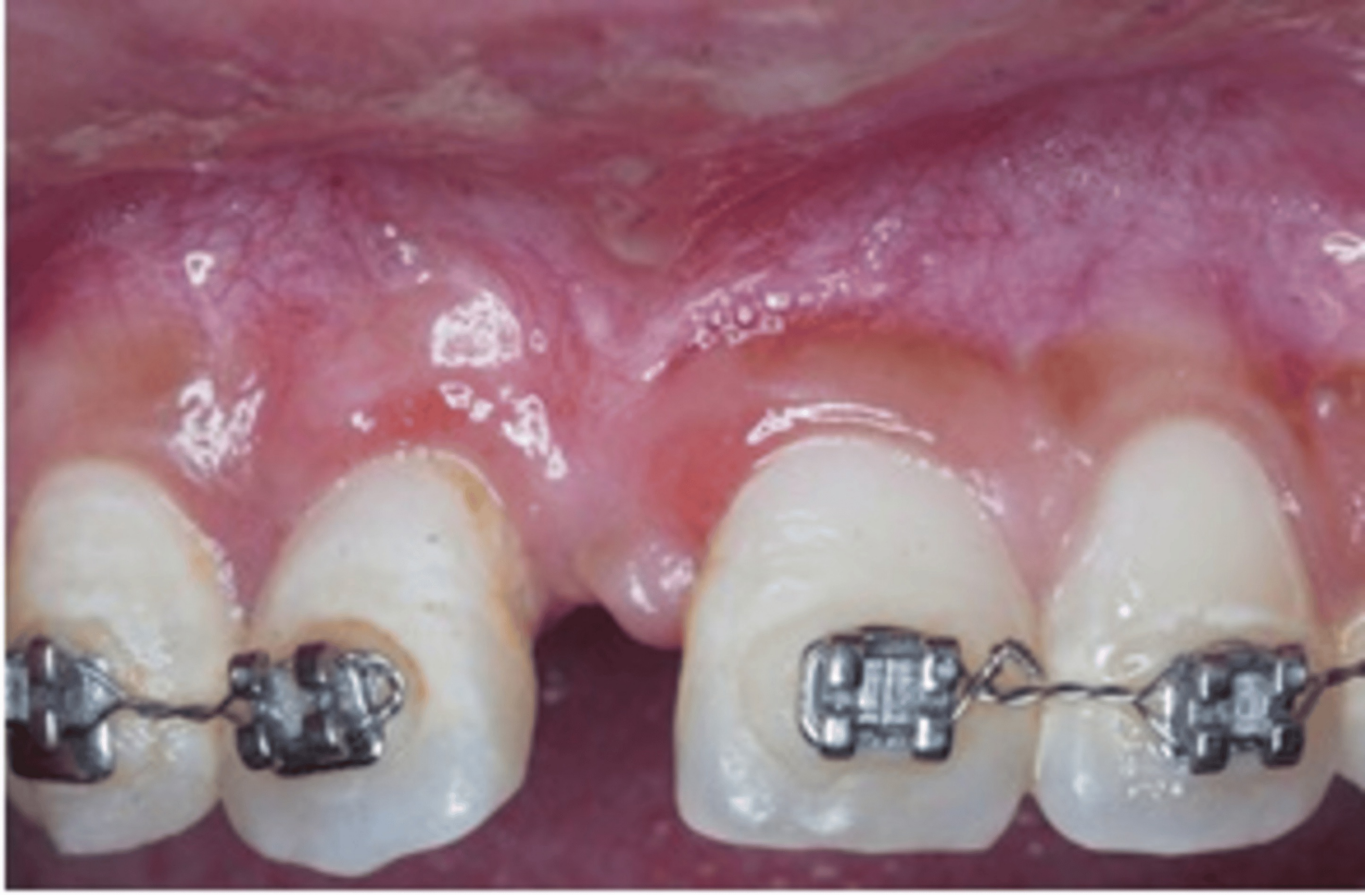
Postoperative photograph shows clinical presentation (6-month post-op).

**Figure 10:**
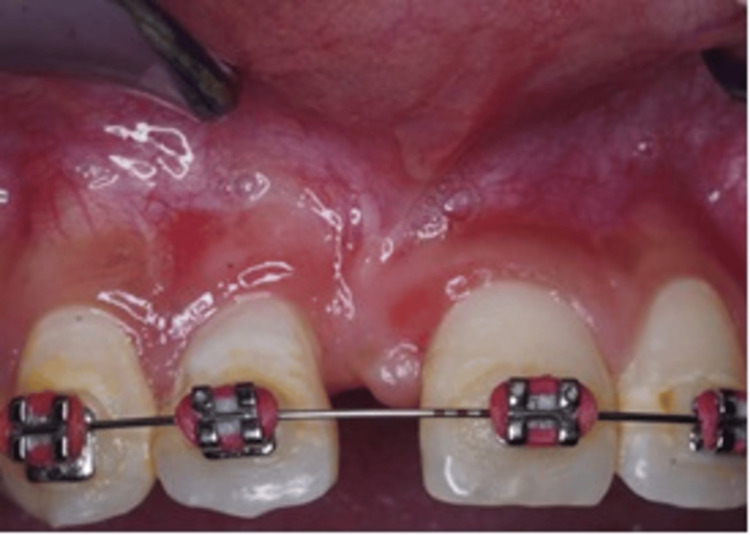
Postoperative photograph shows clinical presentation (1-year post-op).

**Figure 11:**
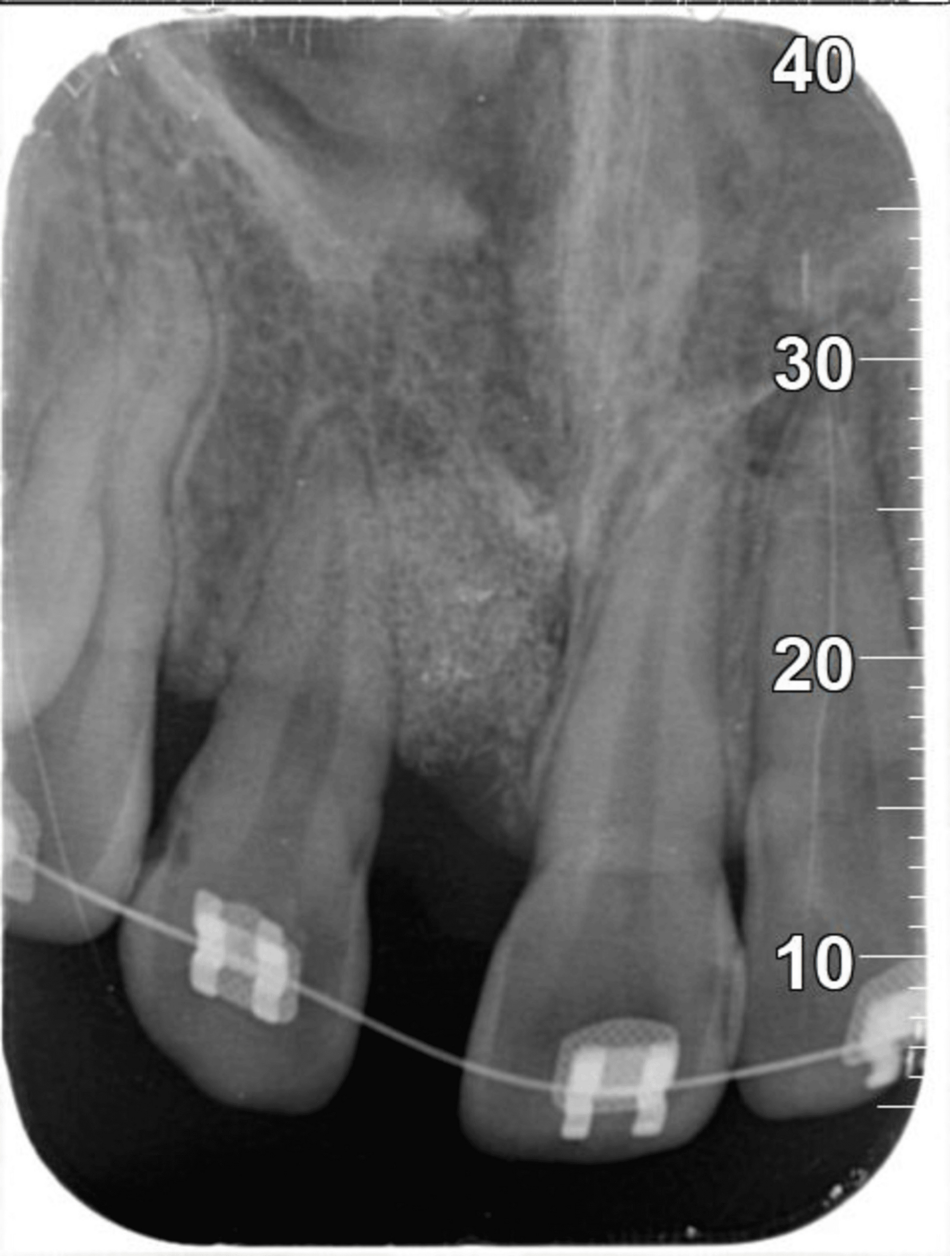
Periapical radiograph revealed increased radiopacity at the surgical site of 11.

**Figure 12:**
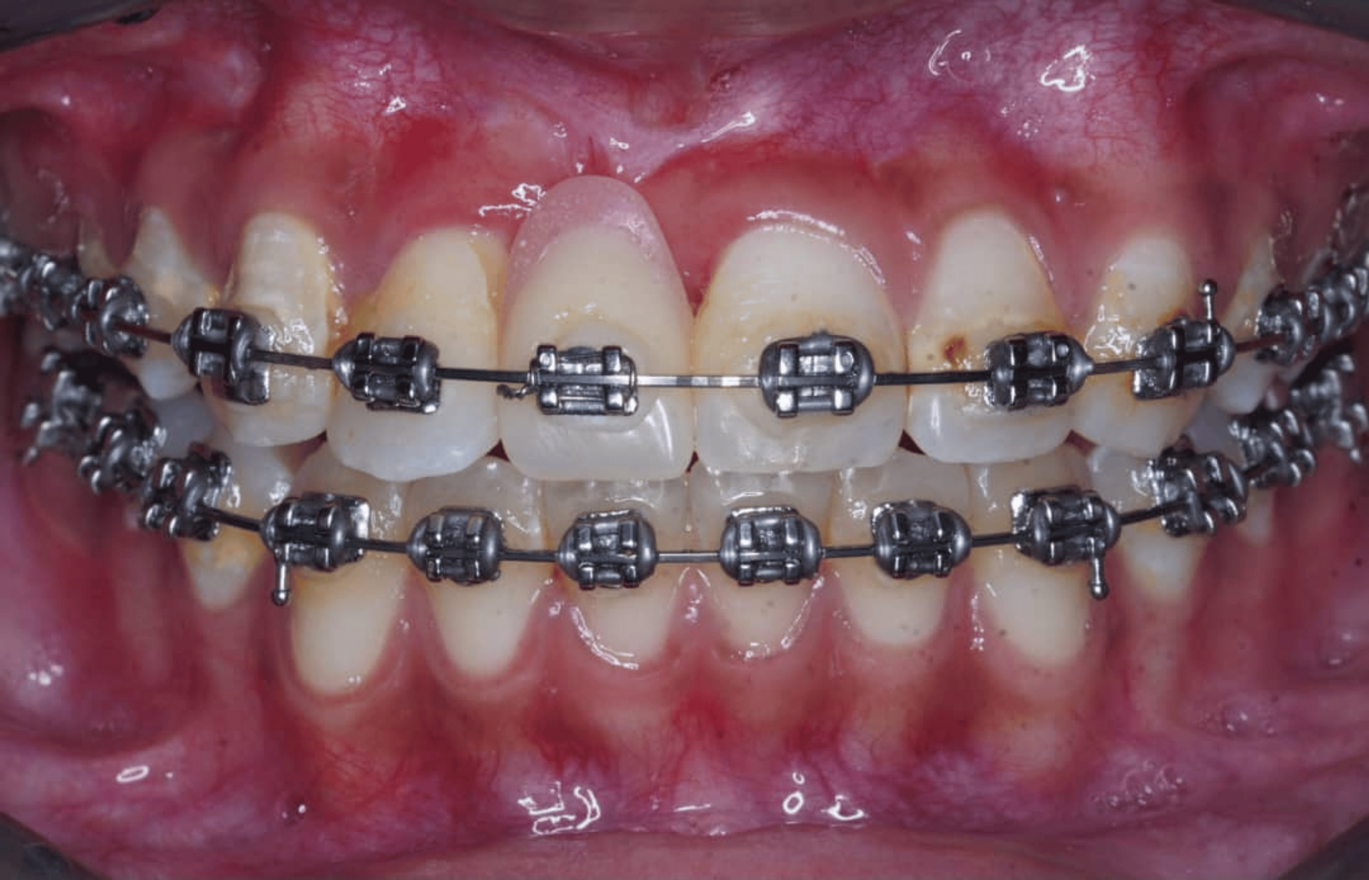
The orthodontist has attached an orthodontic bracket to an acrylic tooth in the 11 area as a temporary replacement.

The patient was reviewed at one week and two weeks (Figures 7-8). During these visits, the wound-healing process was uneventful. Moreover, no side effects or adverse reactions were reported. The patient’s sutures were carefully removed. Orthodontic tooth movement was started six months after regenerative surgery. During the follow-up visits at six months and one year postoperative, increased keratinized tissue was noticed with no gingival recession of tooth 12 (Figures 9-10). The periapical radiograph at one year revealed increased radiopacity at the surgical site (Figure 11). Accordingly, the follow-up visits and the final outcome of the treatment demonstrated the outstanding health of both the hard and soft tissues, emphasizing the effectiveness of the surgical procedure enhanced by GBR and orthodontic treatment. Currently, the patient is undergoing orthodontic treatment to correct the malocclusion. The orthodontist has attached an orthodontic bracket to an acrylic tooth in the 11 area as a temporary replacement (Figure 12).

## Discussion

In this case, the primary objective of GBR was to regenerate both soft and hard tissues to maintain periodontal health in an orthodontic patient. Notably, numerous factors influence the results of periodontal and orthodontic interdisciplinary work, including the method and materials used in regenerative surgery, the type of defect, the timing of orthodontic movement, and the directions of the movement. Utilizing the xenograft bone and ADM membrane to achieve this outcome, these materials provide a supportive framework for new bone formation while stabilizing the soft tissue. Unlike collagen membranes, which can potentially resorb before adequate tissue formation, ADM offers enhanced durability, which is essential for protecting the grafted area over a longer healing period.

Studies have revealed that the selection of membrane can significantly impact the success of GBR in complex cases, particularly in maintaining bone graft stability and preventing soft tissue collapse [5]. Additionally, the frenotomy and deepening of the labial sulcus were performed to alleviate muscle tension and optimize the environment for bone regeneration. Frenotomy, combined with vestibuloplasty, has been demonstrated to create a more favorable setting for graft retention and soft tissue thickening, thus enhancing the long-term stability of bone and soft tissues around the graft site [6].

The selection of materials played a crucial role in this case. Recent advancements in biomaterials for periodontal regeneration, such as the use of biodegradable nanoparticle-in-nanofiber membranes, have presented promising results in enhancing cell adhesion, biocompatibility, and antibacterial properties while maintaining the necessary mechanical properties to support tissue regeneration [7]. In particular, xenograft bone provided a biocompatible scaffold for new bone formation, while the ADM membrane offered several advantages over traditional collagen membranes. The ADM serves a dual purpose in GBR: 1) bone graft containment and 2) soft tissue thickness and stability [8,9]. Additionally, it acts as a barrier to prevent the ingrowth of unwanted soft tissue into the bone graft site. This allows time for the bone graft to mature and integrate properly [10]. Additionally, its robust structure and slower resorption rate compared to some collagen membranes support the overlying soft tissues [11]. This is particularly crucial in areas where maintaining or augmenting soft tissue thickness is crucial for long-term stability and esthetics, which is often a key concern in orthodontic cases. This condition can be observed in this case, which resulted in increased keratinized tissue and no gingival recession of tooth 12.

The timing of orthodontic force application following GBR is a critical factor influencing treatment success. In this case, orthodontic tooth movement was started six months after regenerative surgery. While specific timelines vary depending on the complexity of the defect and individual healing characteristics, Pini Prato and Chombrone (2020) recommended starting orthodontic therapy (OT) six to nine months after surgical periodontal treatment and 12 months after regenerative periodontal surgeries [12]. However, recently The European Federation of Periodontology (EFP) S3-level clinical practice guidelines suggested not to wait for a prolonged healing period since there were comparable outcomes between a short and a prolonged period between periodontal/regenerative and OT [13]. Moreover, a recent study indicated that orthodontic tooth movement may not jeopardize the regenerative effect and may have the potential to improve the overall efficiency of the treatment [14]. According to the study, periodontal regeneration provided a better foundation for orthodontic movement since the orthodontic force plays a positive role in the better turnover rate of bone grafts and enhances the process of bone formation [14].

The present case revealed increased radiopacity at the surgical site following GBR by periapical radiograph, which only provides two-dimensional (2D) images. However, one limitation of this case report is the absence of cone-beam computed tomography (CBCT) imaging to assess the final outcome. CBCT, a reliable and widely used tool, provides a three-dimensional (3D) exploration of periodontal osseous defects [15]. Thus, the changes in the surgical site can be compared accurately using CBCT to evaluate the effectiveness and stability of periodontal treatment.

## Conclusions

In conclusion, this case highlights the effectiveness of a comprehensive approach that integrates GBR, meticulous soft tissue management, and strategic timing of orthodontic treatment to regenerate bone and ensure periodontal stability in an orthodontic patient. Furthermore, long-term follow-up is essential to monitor the stability of the regenerated tissues and ensure successful orthodontic outcomes.
